# BEST—Blockchain-Enabled Secure and Trusted Public Emergency Services for Smart Cities Environment

**DOI:** 10.3390/s22155733

**Published:** 2022-07-31

**Authors:** Bhawana, Sushil Kumar, Rajkumar Singh Rathore, Mufti Mahmud, Omprakash Kaiwartya, Jaime Lloret

**Affiliations:** 1 School of Computer and Systems Sciences, Jawaharlal Nehru University, New Delhi 110067, India; bhawan_scs@jnu.ac.in (B.); skdohare@mail.jnu.ac.in (S.K.); 2 Department of Computer Science, Cardiff School of Technologies, Cardiff Metropolitan University, Cardiff Llandaff Campus, Cardiff CF5 2YB, UK; rsrathore@cardiffmet.ac.uk; 3 Department of Computer Science, Nottingham Trent University, Nottingham NG11 8NS, UK; mufti.mahmud@ntu.ac.uk; 4 Computing and Informatics Research Centre, Nottingham Trent University, Nottingham NG11 8NS, UK; 5 Medical Technologies Innovation Facility, Nottingham Trent University, Nottingham NG11 8NS, UK; 6 Department of Communications, Universitat Politècnica de València, 46022 Valencia, Spain; jlloret@dcom.upv.es

**Keywords:** Internet of Things, blockchain, public emergency service, smart contracts, queue model, reputation model

## Abstract

In the last few years, the Internet of things (IoT) has recently gained attention in developing various smart city applications such as smart healthcare, smart supply chain, smart home, smart grid, etc. The existing literature focuses on the smart healthcare system as a public emergency service (PES) to provide timely treatment to the patient. However, little attention is given to a distributed smart fire brigade system as a PES to protect human life and properties from severe fire damage. The traditional PES are developed on a centralised system, which requires high computation and does not ensure timely service fulfilment. Furthermore, these traditional PESs suffer from a lack of trust, transparency, data integrity, and a single point of failure issue. In this context, this paper proposes a Blockchain-Enabled Secure and Trusted (BEST) framework for PES in the smart city environment. The BEST framework focuses on providing a fire brigade service as a PES to the smart home based on IoT device information to protect it from serious fire damage. Further, we used two edge computing servers, an IoT controller and a service controller. The IoT and service controller are used for local storage and to enhance the data processing speed of PES requests and PES fulfilments, respectively. The IoT controller manages an access control list to keep track of registered IoT gateways and their IoT devices, avoiding misguiding the PES department. The service controller utilised the queue model to handle the PES requests based on the minimum service queue length. Further, various smart contracts are designed on the Hyperledger Fabric platform to automatically call a PES either in the presence or absence of the smart-home owner under uncertain environmental conditions. The performance evaluation of the proposed BEST framework indicates the benefits of utilising the distributed environment and the smart contract logic. The various simulation results are evaluated in terms of service queue length, utilisation, actual arrival time, expected arrival time, number of PES departments, number of PES providers, and end-to-end delay. These simulation results show the effectiveness and feasibility of the BEST framework.

## 1. Introduction

**Figure 1:**
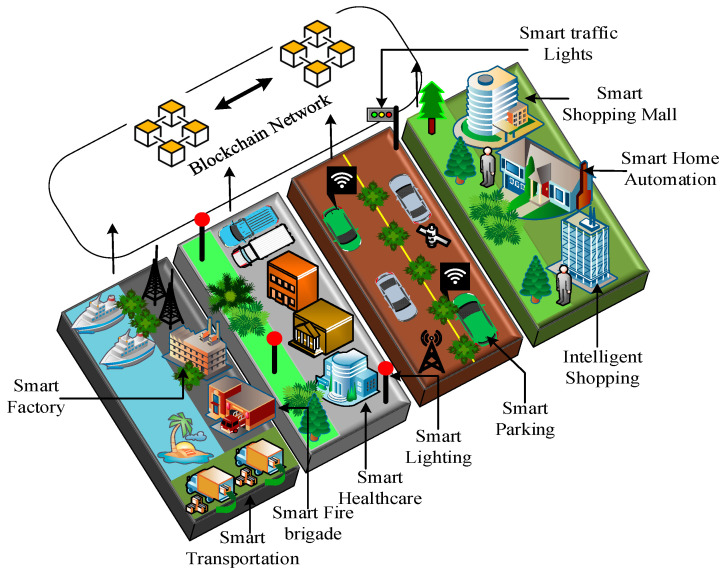
Smart city applications.

The smart city covers urban areas equipped with Internet of Things (IoT) devices [1]. These IoT devices receive surrounding data and provide meaningful information to smart-city people to bring convenience to their day-to-day life [2,3]. The meaningful information is provided to smart-city people through smart city applications. The smart city applications include smart healthcare, smart home automation, smart supply chain, smart grid, smart agriculture, and smart traffic environment [4,5], as shown in Figure 1. Due to the smart city applications, the demand for IoT devices is increasing day by day and is expected to reach 50 billion worldwide by 2030 [6]. The data generated by these increasing number of IoT devices is growing exponentially, and handling such data becomes more challenging. Furthermore, an IoT device has a few limitations, such as low computation power, limited storage, restricted transmission range, and vulnerability to attack [7]. However, few options are currently available to systematically manage these connected IoT devices and transfer the data to the centralised system for further processing [8]. The cluster head is one of the options. Still, it suffers from vast data storage, scalability, and fast information processing. The centralised system has drawbacks, such as single point of failure, trust, transparency, and data integrity. Therefore, the edge computing server and blockchain come into the picture to overcome such problems. The edge computing server resides close to the IoT devices to provide high bandwidth, fast computing, scalability, data storage, and efficiently manage numerous IoT devices [9]. The blockchain adds distributed trust and transparency through a distributed ledger and consensus protocol. 

Another important consideration in the smart city is providing Public Emergency Services (PESs) to the smart-city people to protect them from serious hazards. The existing PESs only focus on the smart healthcare system to timely provide a treatment based on a healthcare service request to the patient based on the centralised system [10]. Similarly, we require other PESs, such as a smart fire brigade system that efficiently handles the fire brigade service request for a smart home in the presence of a fire. The IoT devices are assembled in the smart home to capture the surrounding information and transfer the received information via an edge computing server to the centralised system for further processing [11]. The centralised system provides access to the fire brigade department to fetch the fire brigade service requests to take necessary action. Due to the centralised system, the smart-home owner is unable to verify whether the assigned fire brigade department is properly handling their fire brigade request or not. Additionally, the smart-home owner is unable to track whether their fire brigade service request is processed successfully or still in the waiting queue. Mostly, the centralised system suffers from a single point of failure due to which fire brigade service requests are not processed efficiently, and the connected fire brigade department is unable to access fire brigade service request information on time [12]. Further, the centralised system also does not ensure the arrival of a fire brigade in a minimum waiting time after receiving the fire brigade service request. 

To address the above-raised issues, this paper proposes a Blockchain-Enabled Secure and Trusted (BEST) framework to handle the PES in a smart city environment. The proposed BEST framework identifies the presence of fire in a smart home using IoT devices and provides a fire brigade service as a PES to cure severe fire damage. The main contribution of the paper is described as follows:

1. A three-layered BEST framework architecture is presented, consisting of smart homes, IoT controllers, service controllers, and blockchain nodes.
2. The IoT controller and service controller behave as edge computing servers. The IoT controller manages a set of smart homes that belong to its area and sends a PES request (i.e., fire brigade service request) under uncertain environmental conditions using a smart contract. Furthermore, it manages an access control list to keep the information of registered IoT devices that belong to smart homes to protect the PES department from receiving the wrong information. The service controller controls numerous PES departments (i.e., fire brigade departments) and uses the queue model to balance PES requests fairly among PES departments.
3. The various smart contracts are designed to register the smart home, IoT controller, and service controller. The other smart contract, such as request PES and allocate PES, automatically invokes and handles the PES request on the blockchain network. Further, the service controller generates a final reputation value for a PES department after fulfilling the PES request using smart contract logic on the blockchain network.
4. The proposed BEST framework is designed on the Hyperledger Fabric platform to bring trust and transparency to the overall framework architecture. The simulation results are evaluated in terms of waiting time, utilisation, actual reaching time, expected reaching time, final reputation value, and end-to-end delay to show the effectiveness of the proposed framework.

The rest of the paper is organised as follows. In Section 2, a literature review on the smart city applications based on the blockchain is presented. In Section 3, background knowledge of blockchain technology is provided. A detailed description of the proposed BEST framework and smart contracts are provided in Section 4. Section 5 presents the simulation and results of the BEST framework, followed by the conclusions and future work in Section 6.

## 2. Related work

### 2.1. Blockchain for Smart Cities Environment 

In [13], the authors proposed an IoT-based smart manufacturing system for quality assurance applications. The blockchain is utilised to build a trust relationship and improve security concerns in the manufacturing life cycle process. In [14], the authors presented a lightweight expandable blockchain model for a smart factory application. An access control list is also designed to prevent malicious activities using Bell-La-Padula and Biba models. In [15], the authors proposed a resource utilisation model for IoT devices in the smart city in which edge computing and miner nodes are placed together in the blockchain network. The edge computing node is responsible for proper functioning IoT devices, whereas the miner node performs high computation tasks. In [16], the author proposed a three-tier architecture supporting scalable sharing economy services in a mega smart city. The blockchain nodes synchronise data with the backend cloud, and artificial intelligence identifies the meaning pattern through deep and convolutional neural networks. These patterns are used to share various economic services, depending on the need. In [17], the author proposed a blockchain-based electronic health record system that utilises an identity-based signature scheme using the Diffie–Hellman assumption to authenticate multiple authorities with the electronic health record system. In [18], the authors presented a patient-centric access control system using blockchain to secure health information. Further, a lightweight double encryption algorithm and Diffie–Hellman key exchange are utilised to bring anonymity and authenticity to the proposed system. In [19], the authors presented a blockchain-based electronic health record sharing protocol that used the proof of authentication consensus protocol. The health record sharing protocol achieves privacy through key search encryption and a proxy re-encryption technique. In [20], the authors developed an automatic medical insurance claims service system using blockchain and smart contracts to solve risk control and anti-money laundering problems. In [21], the authors proposed a lightweight access control system for an IoT network using blockchain in which access control policies are designed for IoT devices to permit access. 

### 2.2. Blockchain for Emergency Services

A blockchain-enabled emergency service architecture is suggested for smart homes, which ensures security and privacy authentication mechanisms [22]. In [23], the authors designed a private blockchain-based access control model for the smart home to protect against illegal access. Further, two-way secure authentication and token-based access control policies are proposed to grant the service provider access to IoT devices in the smart home. In [24], the authors presented a blockchain-enabled remote user authentication system for the smart home in which authentication is performed using a group of signature and message authentication code techniques. In [25], the authors presented an intelligent agriculture system based on blockchain in which a hash-based message authentication code is utilised to determine the message authenticity. In [26], the authors proposed a blockchain-enabled secure firmware framework for managing heterogeneous devices that keeps track of firmware update history. In [27], the author designed a microgrid architecture using blockchain for the smart energy grid to buy and sell energy between energy supplier and consumer. In [28], the authors proposed a decentralised hybrid peer-to-peer energy trading system with a bidding mechanism using blockchain. The system enables smart homes with renewable resources to trade energy for other consumers to reduce dependency on the utility grid during peak hours. The blockchain eliminates the need for central authority by managing distributed energy transactions. In [29], the authors proposed a peer crop insurance framework for farmers using blockchain to cover only one type of risk, i.e., excessive rainfall. In [30], the authors presented a blockchain-based smart contract framework for the drought insurance system to explain the mechanism for crop insurance. In [31], the authors proposed a blockchain-based framework for auto-insurance claims in which automated vehicles utilise sensors to share information. In [32], the authors proposed a vehicle insurance system using blockchain to record vehicle insurance information, which acts as evidence during disputes. After analysing the existing work on blockchain-based smart city applications, it is identified that researchers are mostly focusing on smart home automation applications, smart healthcare applications, smart insurance applications, and smart grid applications. In the smart home automation application, the authors used the access control list information to provide access to the smart objects in the smart home. In smart healthcare applications, the authors add security to the electronic health record using various security mechanisms with the integration of blockchain to prevent unauthorised access. Furthermore, they provide emergency services such as ambulances using blockchain to timely provide healthcare services to the patient. The authors build various blockchain-based insurance systems in a smart insurance system to protect the insurer from fraud. Finally, the authors considered the energy trading mechanism between the producers and consumers using blockchain in smart grid applications. Most authors considered the public blockchain platform to build the smart city application, which requires high computation resources to add a block to the blockchain network. Furthermore, a public blockchain platform requires a transaction fee cost associated with each transaction to finalise a transaction in the blockchain network. Designing a fire brigade service application using blockchain to instantly provide a fire brigade service to the smart home under undetermined environmental conditions to protect a smart home from serious fire damage is still an open challenge. We designed a public emergency service system using a private blockchain for smart homes to address this issue. 

## 3. Preliminaries on Blockchain

The term blockchain was popularised from Bitcoin in 2008 [33] by an unknown person named Satoshi Nakamoto. The blockchain is an append-only data structure to maintain immutable transactions in the form of a distributed ledger among untrusted and unknown individuals to replace the centralised system. Bitcoin is a cryptocurrency, whereas the blockchain is the underlying technology. The Bitcoin blockchain has become famous for the financial application in which individuals transfer digital assets from one location to another in a few minutes. The individuals in the Bitcoin blockchain are connected in a peer-to-peer network and use a pair of public/private keys to sign a transaction. The success of the Bitcoin blockchain is the proof-of-work-based consensus algorithm, which eliminates the intermediate role of confirming the transaction on the Bitcoin blockchain network. Furthermore, the longest chain rule mechanism in the Bitcoin blockchain handles the double spend problem. The unique properties of blockchain technology, such as immutability, transparency, distributed ledger, consensus protocol, block, and smart contracts, gained the attention of various researchers and companies. Later, researchers and companies started exploring different opportunities and came up with two types of blockchain platforms: public and private. The public blockchain platform allows anyone to become a part of the blockchain network, requiring only a public/private key and a wallet address. The famous public blockchain platforms are Ethereum, R3 Corda, Litecoin, Quorum, etc. In comparison, the private blockchain platform only allows known persons to become a part of the private blockchain. The most known private blockchain platforms are Hyperledger Fabric, Hyperledger Besu, Hyperledger Indy, etc. These two blockchain platforms are used to implement financial applications and develop smart city applications.

### 3.1. Smart Contract

Nick Szabo proposed the concept of a smart contract in 1994, in which he explained that a smart contract is a self-executable code used to write a business logic or agreement between two or more parties [34]. The definition of the smart contract changed after the invention of blockchain technology. According to the blockchain, a smart contract is a Turing complete logic used to write any application logic stored at a permanent address in the blockchain network to roll out the involvement of any third party [35]. Once the smart contract is deployed in the blockchain network, it cannot be modified and executes automatically when certain conditions meet. 

### 3.2. Block 

**Figure 2:**
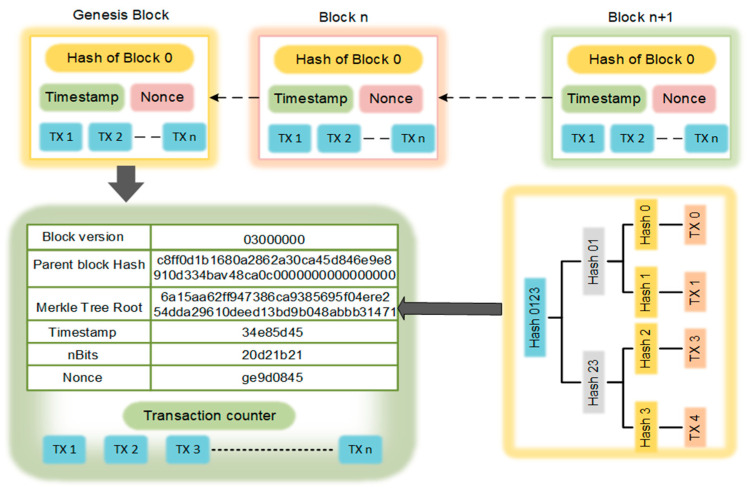
Block structure.

The block is an essential component in the blockchain network, which consists of a block header and a block body, as shown in Figure 2. The block body contains the application-specific transaction information. The block header manages a block version, parent block hash, Merkle tree root hash, timestamp, nBits, and a nonce [36]. The Merkle root hash contains a single hash corresponding to all transactions available in a block. The first block in the blockchain network is called the genesis block. The genesis block holds the information of the validator or miner, consensus protocol, and smart contract logic address. The upcoming blocks in the blockchain network build on top of the genesis block linked together through a cryptographic hash function. 

### 3.3. Private Blockchain

The Hyperledger Fabric is a famous private blockchain platform hosted by the Hyperledger Foundation in 2015 [37]. To understand the working of the proposed BEST framework, a description of the Hyperledger Fabric components is explained as follows:

(a) Membership service provider: The MSP provides a digital certificate to the Certificate Authority (CA) in the private blockchain network. The MSP keeps the information of generated digital certificates in a certificate list to authenticate a CA when required. The MSP also gives authority to the CA to distribute digital certificates within its organisation.
(b) Certificate authority: The CA resides within an organisation and provides digital certificates during the creation of peers and a client.
(c) Peer: The peer is categorised into two types: endorsing peer and committing peer. The endorsing peer performs a transaction endorsement to achieve the consensus in the private blockchain network. In contrast, the committing peer validates and manages a set of transactions through a block in the private blockchain network.
(d) Client: The client interacts with the private blockchain network using smart contracts to generate transactions.
(e) Orderer: The orderer bundles the endorsed transactions and arranges them in a timestamped order to create a valid block in the private blockchain network and broadcast it to the committing peer.
(f) Channel: The channel is a medium to connect multiple organisations to receive the same set of transaction information in the private blockchain network to manage consistency.

## 4. BEST—Blockchain-Enabled Secure and Trusted Framework for Public Emergency Services 

This section is divided into four parts. The proposed BEST framework’s system architecture is presented in the first part. In the second and third parts, a queue model is designed to select a PES department for a smart home and a reputation model for a PES department after fulfilling a PES request. The fourth part presents a network setup for the private blockchain network and the implementation of various smart contracts. The working of each part is explained as follows and the abbreviations used in designing the queue and reputation model, as shown in Table 1. 

### 4.1. System Architecture 

**Figure 3:**
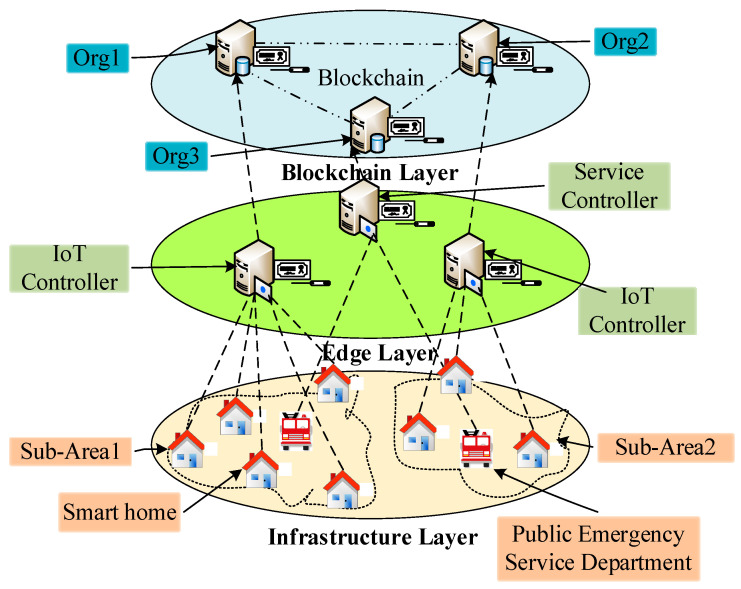
The system architecture of the BEST framework.

The system architecture of the BEST framework comprises three layers, as shown in Figure 3. The infrastructure layer is divided into several sub-areas in which each sub-area consists of several smart homes and a PES department (i.e., fire brigade department). The smart home is equipped with IoT devices such as a temperature device, a smoke device, a humidity device, a fire alarm, and an IoT gateway. The PES department manages multiple PES providers (i.e., fire brigades). Each PES department maintains a service queue to handle the PES requests instantly. Further, the edge layer comprises IoT controllers and a service controller. The IoT controller manages the IoT gateway and IoT device information in its area. The IoT controller continuously monitors the IoT device data received from an IoT gateway and takes the necessary action when IoT device data reach the threshold. Furthermore, the IoT controller keeps track of deployed IoT devices in a smart home using an access control list to detect the placement of any malicious IoT device quickly. The service controller stores the information of multiple PES departments located in various areas with their service queue length. Further, the service controller runs a queueing model to select an appropriate PES department for a smart home. The blockchain layer is a collection of Fabric organisations. Each Fabric organisation is associated with an IoT or service controller. Further, each Fabric organisation stores the smart contract and blockchain information in the form of a distributed ledger. It is clarified that the three-layer blockchain architectural workflow of the proposed BEST framework considers IoT gateways and nodes in the edge layer. Tempering of data in the edge layer is addressed in the upper blockchain layer in the framework, which eliminates node-based data tempering risks.

### 4.2. Queue Model for Public Emergency Service Department

**Figure 4:**
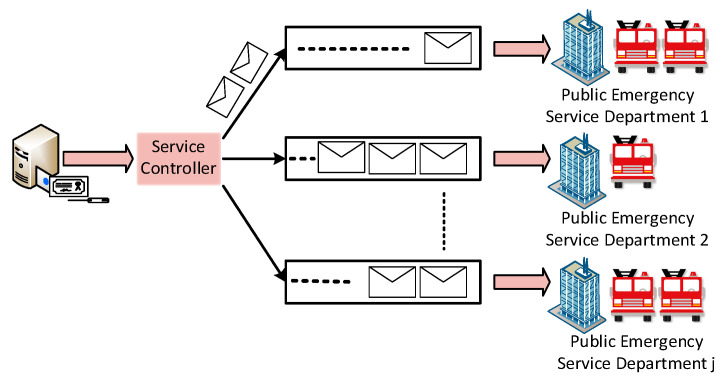
Queue model for the BEST framework.

The service controller uses the queue model to select an appropriate PES department with a minimum service queue length for a smart home that handles a PES request, as shown in Figure 4. For instance, a classical M/M/c queueing theory model is utilised to design the queue model for the proposed BEST framework in which PES requests follow the first come, first serve queueing discipline [38,39,40]. Based on Kendall’s notation [41], the first and second M indicate the arrival and service time for the PES request. The arrival and service time for the PES request follow the Markovian Exponential distribution. Further, the c denotes the number of PES departments in the BEST framework. The waiting time to confirm the PES request for a smart home depends on two parameters. The first parameter is the local computation of a service controller to select a suitable PES department based on its service queue length, and the second is the arrival time of the PES request and service rate of the PES department, which indirectly depends on the number of PES providers. The utilization $\;U_{j}^{PESD}\;$of the$\;j^{\mathrm{th}}\;$PES department, where$\;j\in 1,2,\ldots ,N^{PESD},$ can be expressed as given by Equation (1).

(1) $$U_{j}^{PESD}=\frac{\lambda_{j}^{PESD}}{\mu_{j}^{PESD}}$$

where $\lambda_{j}^{PESD}\;$and $\mu_{j}^{PESD}\;$represent the arrival time of PES requests and the service rate of the$\;j^{\mathrm{th}\;}$PES department, respectively. 

The service queue length$\;SQL_{j}^{PESD}\;$for the$\;j^{\mathrm{th}}\;$PES department can be expressed as given by Equations (2) and (3).

(2) $$SQL_{j}^{PESD}=\frac{PI_{j}^{PESD}\times {\left(U_{j}^{PESD}\right)}^{N^{PESD}}\times \left(U_{j}^{PESD}\right)}{N^{PESD}!\times {\left(1-U_{j}^{PESD}\right)}^{2}}$$

(3) $$PI_{j}^{PESD}=\frac{1}{\left[\sum_{i=0}^{N^{PESD}-1}\frac{{\left(N^{PESD}\times U_{j}^{PESD}\right)}^{i}}{j!}+\frac{{\left(N^{PESD}\times U_{j}^{PESD}\right)}^{N^{PESD}}}{N^{PESD}!\times \left(1-U_{j}^{PESD}\right)}\right]}$$

where$\;PI_{j}^{PESD}\;$and$\;N^{PESD}\;$indicate the probability of idleness of the$\;j^{\mathrm{th}}\;$PES department and the total number of PES departments, respectively.

The waiting time$\;WT_{j,i}^{PESR}\;$of$\;i^{\mathrm{th}}\;$smart home’s PES request at the selected$\;j^{\mathrm{th}}\;$PES department before receiving the confirmation, where $\;i\in 1,2,\ldots ,\;N^{SH}$, can be expressed as given by Equation (4).

(4) $$WT_{j,i}^{PESR}=\frac{SQL_{j}^{PESD}}{U_{j}^{PESD}}$$

The working of the queue model to select a PES department for a smart home in the BEST framework is given in Algorithm 1 and described as follows: 

Step 1: The $\;p^{\mathrm{th}}\;$ IoT controller continuously monitors the IoT device information of the$\;i^{\mathrm{th}}\;$smart home, which resides under its sub-area, where$\;p\in 1,2,\ldots ,\;N^{IC}$. The IoT controller sets the threshold value corresponding to the temperature device $\;Th^{\alpha }$, smoke device $\;Th^{\beta }$ and humidity device $\;Th^{\gamma }$ to identify the presence of fire in a smart home. Once the IoT device values of the$\;i^{\mathrm{th}}\;$smart home reaches the threshold, the $p^{\mathrm{th}}\;$ IoT controller generates a PES request for that smart home on the private blockchain network. 

Step 2: The service controller receives the PES request of the$\;i^{\mathrm{th}}\;$smart home from the private blockchain network. The service controller retrieves the sub-area $SA_{i}^{SH}$ of the$\;i^{\mathrm{th}}\;$smart home and all PES department sub-areas $\;SA_{j}^{PESD}$. Further, two cases are considered to handle the PES request of the $i^{\mathrm{th}}\;$smart home and are explained as follows: 

Case 1: For instance, the sub-area of the $i^{\mathrm{th}}\;$ smart home and the $j^{\mathrm{th}}\;$ PES department are the same, and the service queue length of $\;SQL_{j}^{PESD}\;$ of the $j^{\mathrm{th}}\;$ PES department is shorter than the other PES departments. The service controller selects the $\;j^{\mathrm{th}}\;$ PES department and forwards the PES request of $i^{\mathrm{th}}\;$ smart home to the selected $\;j^{\mathrm{th}}\;$ PES department on the private blockchain network. 

Case 2: For instance, the sub-area of the $i^{\mathrm{th}}\;$smart home and the $j^{\mathrm{th}}\;$PES department is the same, and the service queue length $\;SQL_{j}^{PESD}\;$ of the $\;j^{\mathrm{th}}\;$ PES department is longer than the other PES departments. The service controller compares the service queue length of other PES departments that belong to different sub-areas and selects the one with the minimum service queue length. The selected $\;j^{\mathrm{th}}\;$ PES department receives a PES request of the $i^{\mathrm{th}}\;$smart home through the service controller on the private blockchain network. 

Step 3: The service controller calls a smart contract on the private blockchain network to confirm the arrival of the PES provider of the selected $j^{\mathrm{th}}\;$ PES department at the$\;i^{\mathrm{th}}\;$smart home location. 

Step 4: After fulfilment of a PES request, the $p^{\mathrm{th}}\;$ IoT controller generates a reputation value on behalf of the $i^{\mathrm{th}}\;$smart home and sends it to the private blockchain network. The reputation value indicates a satisfaction level corresponding to the $j^{\mathrm{th}}\;$ PES department that provides a PES.
**Algorithm 1**: Queue model for PES departments**Input**: Threshold values:$\;\;Th^{\alpha },Th^{\beta },Th^{\gamma }$; service queue length:$\;SQL_{j}^{PESD}$; smart home sub-area:$\;SA_{i}^{SH}$; PES department sub-area:$\;SA_{i}^{PESD}$; **Output**: Select the PES department; **Begin** **For** *p* = 1 to $\;N^{IC}$      **For** *i* = 1 to $N^{SH}$            **If**(temperature$\geq \;Th^{\alpha }$ && smoke$\geq Th^{\beta }$ && humidity$\geq Th^{\gamma }$)                  $p^{\mathrm{th}}$ IoT controller call smart contract;             **Else**                 Record the IoT device data;             **End If**      **End For** **End For** **For**
*j* = 1 to$\;N^{PESD}$      Service controller evaluates $U_{j}^{PESD}\;$ and $\;SQL_{j}^{PESD}$;       Retrieve sub-area information;       **For**
*i* = 1 to $N^{SH}$           **If**($SA_{i}^{PESD}==SA_{i}^{SH})$
                 If($SQL_{j}^{PESD}==minimum\;queue\;length)$
                         Select $j^{\mathrm{th}}$ ESP of same sub-area;                  **Else**
                         Select $j^{\mathrm{th}}$ ESP of different sub-area;                  **End If**            **Else**
                 Do nothing;           **End If**       **End For** **End For** **End**

### 4.3. Reputation Model for Public Emergency Service Department

A simple reputation model is used to evaluate a reputation value for the PES department after fulfilling the PES request for a smart home in the BEST framework [42]. The multiple generated reputation values for a PES department are further utilised to calculate a final reputation value for the same PES department. The final reputation value helps the PES department to view its rank and take the necessary actions in the future to improve its performance. The working to obtain a reputation value for a PES department by a smart home is given in Algorithm 2 and explained as follows: 

Step 1: Initially, evaluate the distance $D_{j,i}\;$between the selected$\;j^{\mathrm{th}}\;$PES department and the$\;i^{\mathrm{th}}\;$smart home can be expressed by Equation (5).

(5) $$D_{j,i}=\sqrt{{\left(X_{i}^{SH}-X_{j}^{PESD}\right)}^{2}+{\left(Y_{i}^{SH}-Y_{j}^{PESD}\right)}^{2}}$$

where $X_{i}^{SH},Y_{i}^{SH}\;$and $X_{j}^{PESD},Y_{j}^{PESD}\;$indicate the location coordinates of the $i^{\mathrm{th}}\;$smart home that requests a PES and the$\;j^{\mathrm{th}}\;$PES department that provides a PES, respectively. 

Step 2: Evaluating the reaching time $RT_{j,i}\;$for the selected $j^{\mathrm{th}}\;$PES department to reach at the$\;i^{\mathrm{th}}\;$smart home location can be expressed by Equation (6).

(6) $$RT_{j,i}=\frac{D_{j,i}}{AS_{j}^{PESD}}$$

where$\;AS_{j}^{PESD}\;$represents the average speed of the selected$\;j^{\mathrm{th}}\;$PES department. 

Step 3: Evaluating the reputation value$\;RV_{j,i}\;$ for the selected $j^{\mathrm{th}}\;$PES department generated by the$\;i^{\mathrm{th}}\;$smart home can be expressed by Equation (7).

(7) $$RV_{j,i}=b+e^{-\gamma .D_{j,i}}$$

where b  and γ are two parameters that control the lower bound and change in rate for reputation value, respectively.

Step 4: Evaluating the expected reaching time$\;ERT_{j,i}\;$for the$\;j^{\mathrm{th}}\;$PES department at the$\;i^{\mathrm{th}}\;$smart home location can be expressed by Equation (8).

(8) $$ERT_{j,i}=WT_{j,i}^{PESR}+RT_{j,i}+T_{j,i}$$

where $WT_{j,i}^{PESR}\;$represents the waiting time of the$\;i^{\mathrm{th}}\;$smart home PES request in the$\;j^{\mathrm{th}}\;$PES department before receiving the confirmation of a PES service provider. The$\;T_{i,j}\;$indicates a time duration taken by the$\;j^{\mathrm{th}}\;$PES department for travelling to reach the$\;i^{\mathrm{th}}\;$smart home location in high traffic. 

Step 5: The calculated expected reaching time$\;ERT_{j,i}\;$of the$\;j^{\mathrm{th}}\;$PES department is attached while sending a confirmation of the PES provider on the private blockchain network. 

Step 6: Once the PES provider of the$\;j^{\mathrm{th}}\;$PES department reaches the$\;i^{\mathrm{th}}\;$smart home location, an actual reaching time$\;ART_{j,i}\;$is evaluated. The actual reaching time is evaluated by varying the time duration$\;T_{j,i}\;$value using Equation (8). Further, to generate a reputation value for the PES department, we considered two cases, which are described as follows: 

Case 1: In-time PES: The first case is named in-time PES, in which the actual reaching time$\;ART_{i,j}\;$is compared with the expected reaching time $\;ERT_{j,i}\;$of the$\;j^{\mathrm{th}}\;$PES department. For instance, the actual reaching time is shorter than the expected reaching time, and the$\;i^{\mathrm{th}}\;$smart home generates a positive reputation value $\;PRV_{j,i}\;$for the$j^{\mathrm{th}}\;$PES department, which can be expressed as given by Equation (9).

(9) $$PRV_{j,i}=PRV_{j,i}+\left(RV_{j,i}\times \left(+1\right)\right)$$

Case 2: Delayed PES: The second case is called delayed PES. For instance, the actual reaching time is greater than the expected reaching time, and the$\;i^{\mathrm{th}}\;$smart home generates a negative reputation value $\;NRV_{j,i}\;$for $j^{\mathrm{th}}\;$PES department, which can be expressed as given by Equation (10).

(10) $$NRV_{j,i}=NRV_{j,i}+\left(RV_{j,i}\times \left(-1\right)\right)$$

Step 7: The service controller obtains a final reputation value $FRV_{j}\;$for the$\;j^{\mathrm{th}}\;$PES department using Equations (9) and (10), which can be expressed as given by Equation (11).

(11) $$FRV_{j}=\overset{N^{PESD}}{\underset{j=1}{\sum }}\left[\overset{T}{\underset{t=0}{\sum }}\left(\overset{N^{SH}}{\underset{i=1}{\sum }}PRV_{j,i}\right)+\overset{T}{\underset{t=0}{\sum }}\left(\overset{N^{SH}}{\underset{i=1}{\sum }}NRV_{j,i}\right)\right]$$

where T represents the time interval of twenty-four hours. $\;N^{SH}\;$and$\;N^{PESD}\;$indicate the total number of smart homes and PES departments, respectively.

Step 8: The service controller uploads the final reputation value $\;FRV_{j}\;$of the $j^{\mathrm{th}}\;$PES department onto the private blockchain network.
**Algorithm 2**: Reputation model for PES departments.Input: Smart home location: $X_{i}^{SH},Y_{i}^{SH}$; PES department location: $X_{j}^{PESD},Y_{j}^{PESD}$; Expected reaching time: $ERT_{j,i}$; Average speed: $\;AS_{j}^{PESD}$; Output: Final reputation value for the PES department **Begin** **For** *j* = 1 to$\;N^{PESD}$   **For** *i* = 1 to $N^{SH}$      Evaluate $D_{j,i}$; $RT_{j,i}$; $RV_{j,i}$;$\;ERT_{j,i}$;$\;ART_{j,i}$;      **If**($ART_{j,i}\leq ERT_{j,i}$)           Evaluate $PRV_{j,i}$;       **Else**           Evaluate $NRV_{j,i}$      **End If**
   **End For** Evaluate $FRV_{j}$; **End For** **End**

### 4.4. Working of Private Blockchain Network

The working of the private blockchain network consists of two parts: the setup of a private blockchain network for the proposed BEST framework and the smart contract’s functionality to call PES requests on the private blockchain network. 

#### 4.4.1. Private Blockchain Network Setup 

**Figure 5:**
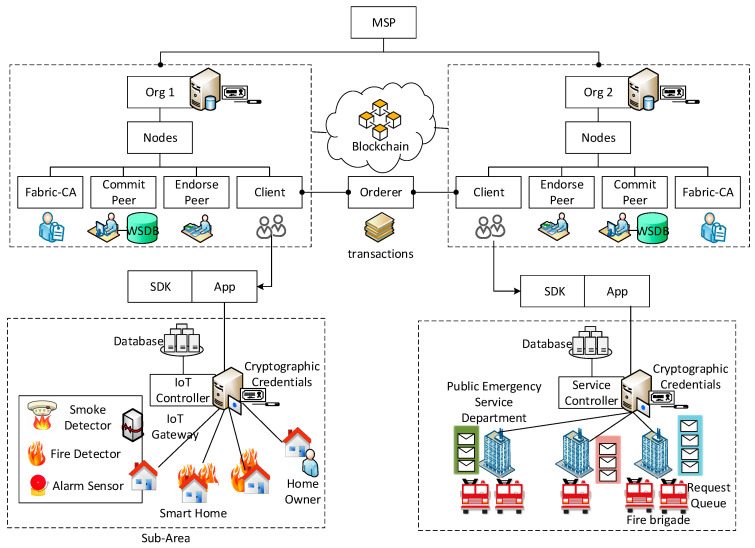
Functional architecture of the BEST framework.

Initially, an administrator (i.e., a government organisation) sets up the private blockchain network on the Hyperledger Fabric platform and deploys the smart contracts to perform various blockchain-related operations in the BEST framework, as shown in Figure 5. An administrator separately creates a Fabric organisation corresponding to each IoT controller $IoT^{C}\;$and a service controller$\;S^{C}\;$represented as$\;Org^{F}$. The private blockchain network consists of$\;N^{Org^{F}}\;$number of Fabric organisations, where$\;N^{Org^{F}}\in \;Org_{1}^{F},\;Org_{2}^{F},\ldots ,Org_{n}^{F}$. The MSP creates a digital certificate $Cert\_Org_{CA}^{F}\;$for each CA presented inside a Fabric organisation to make a Fabric organisation valid on the private blockchain network, which can be expressed as given by Equation (12).

(12) $$MSP\overset{generate}{\to }\left\{Cert\_Org_{CA_{1}}^{F},\;Cert\_Org_{CA_{2}}^{F},\ldots ,\;Cert\_Org_{CA_{n}}^{F}\right\}$$

where $Cert\_Org_{CA_{n}}^{F}\;$represents the digital certificate of the $n^{\mathrm{th}}\;$CA. After receiving digital certificates from the MSP, the CA generates digital certificates for peers present inside the Fabric organisation, which can be expressed as given by Equation (13).

(13) $$Org_{CA}^{F}\overset{generate}{\to }\left\{Cert\_Org_{peer_{1}}^{F},\;Cert\_Org_{peer_{2}}^{F},\ldots ,\;Cert\_Org_{peer_{n}}^{F}\right\}$$

where $Cert\_Org_{peer_{n}}^{F}\;$indicates the digital certificate of the$\;n^{\mathrm{th}}\;$peer in the Fabric organisation. It is assumed that a Fabric organisation may contain multiple peers. Once the CA generates all the digital certificates in its Fabric organisation, the CA connects its Fabric organisation with a common channel, which can be expressed as given by Equation (14).

(14) $$\left\{Org_{1}^{F},\;Org_{2}^{F},\ldots ,Org_{n}^{F}\right\}\;\overset{connect}{\to }\;Channel$$

Thus far, the private blockchain network is established. Now, the trusted authority installs smart contracts on all Fabric organisation peers and channels through the software development kit, which can be expressed as given by Equation (15).

(15) $$Install\;Smart\;Contract\overset{SDK}{\to }\left\{Org_{peer_{1}}^{F},\;Org_{peer_{2}}^{F},\ldots ,\;Org_{peer_{n}}^{F},channel\right\}$$

As explained below, the IoT and service controllers can interact with the deployed smart contracts.

#### 4.4.2. Smart Contracts 

The functioning of various smart contracts for the proposed BEST frameworks is given in Algorithm 3 and explained as follows:
**Algorithm 3**: Smart Contracts **Begin** **For** *p* = 1 to$\;N^{IC}$
      Call register_IC smart contract;       Enter necessary details;       Receive $PK_{p}^{IoT^{C}},SK_{p}^{IoT^{C}}$; **End For** **For** *i* = 1 to$\;N^{SH}$       **For** *p* = 1 to 1 to$\;N^{IC}$           **If**($\;SA_{i}^{SH}==SA_{p}^{IC}$)                Call API of register_SH
               Enter necessary details;                Receive$\;PK_{i}^{SH^{IoT\_G}},SK_{i}^{SH^{IoT\_G}}$;           **Else**
               Do nothing;           **End If**      **End For** **End For**
**For** *j* = 1 to$\;N^{PESD}$
      Call register_PESD smart contract;       Enter necessary details;       Receive $PK_{j}^{PESD},\;SK_{j}^{PESD}$; **End For** **For** *i* = 1 to$\;N^{SH}$
     Call API of call_PES_servicProvider smart contract;      Enter necessary details;      Call Algorithm 1 **End For**
**For** *p* = 1 to 1 to$\;N^{IC}$
      Call reputationGeneration_PESD smart contract;       Enter necessary detail;       Call Algorithm 2; **End For** **For** *j* = 1 to$\;N^{PESD}$
      Service controller Call finalReputationUpdation_PESD smart contract;       Call Algorithm 2        Return$\;FRV_{j}^{PESD}$ **End For** **End**

##### Registration of IoT Controller Smart Contract

Step 1: The$\;p^{\mathrm{th}}\;$IoT controller calls upon the register IoT controller (register_IC) smart contract function through a client to become a legitimate blockchain node. To complete the registration process, the $p^{\mathrm{th}}\;$IoT controller passes the required information, including IoT controller valid identity$\;ID_{p}^{IoT^{C}}$ and sub-area $SA_{p}^{IoT^{C}}$, which can be expressed as given by Equation (16).

(16) $$register\_IC=<ID_{p}^{IoT^{C}}\|SA_{p}^{IoT^{C}}\|timestamp>$$

Step 2: The endorsing peer receives the$\;p^{\mathrm{th}}\;$IoT controller registration request and process. The endorsing peer checks the provided information and uses its digital certificate $Cert\_Org_{peer}^{F}\;$to sign the registration request and send it back to the client using the blockchain transaction Tx, which can be expressed as given by Equation (17).

(17) $$Tx=Cert\_Org_{peer}^{F}<register\_IC\|timestamp>$$

The client collects the signed transaction and forwards it to the orderer. The orderer verifies the collected suitable number of transactions and broadcasts a new block of valid transactions to committing peers of every Fabric organisation. This can be expressed as given by Equation (18).

(18) $$Block=Orderer<Tx^{ID}\left\|register\_IC\right\|Cert\_Org_{peer}^{F}\|timestamp>$$

where $Tx^{ID}$ and the timestamp represent the identity and timestamp of the transaction.

Step 3: The committing peer informs the client of successful registration and generates a pair of public/private keys $PK_{p}^{IoT^{C}},SK_{p}^{IoT^{C}}\;$for the $p^{\mathrm{th}}\;$IoT controller. The public key is used to uniquely identify the $p^{\mathrm{th}}\;$IoT controller on the private blockchain network. 

##### Registration of Service Controller Smart Contract

Step 1: The service controller invokes the register service controller (register_SC) smart contract function via a client. The service controller provides necessary information for completing registration, such as a valid identity$\;ID^{S^{C}}$, category (i.e., fire brigade as PES), and predetermined threshold values$\;(Th^{\alpha },Th^{\beta },\;Th^{\gamma })$, which can be expressed as given in Equation (19).

(19) $$register\_SC=<S^{S^{C}}\|\;category\|timestamp\|Th^{\alpha },Th^{\beta },Th^{\gamma }>$$

Step 2: The endorsing peer collects the registration request and signs the registration request using its digital signature$\;Cert\_Org_{peer}^{F}$. The endorsing peer returns the signed registration request to the client through Tx, which can be expressed as given by Equation (20).

(20) $$Tx=Cert\_Org_{peer}^{F}<register\_SC\|timestamp>$$

The client receives the signed transaction and sends it to the orderer. The orderer checks received transactions and broadcasts a new block to the committing peer to update their distributed ledger with updated information, which can be expressed as given by Equation (21).

(21) $$Block=Orderer<Tx^{ID}\|register\_SC\|Cert\_Org_{peer}^{F}\|timestamp>$$

Step 3: The committing peer updates the client and obtains a pair of public/private keys $PK^{S^{C}},SK^{S^{C}}\;$for the service controller. The public key uniquely identifies the service controller on the private blockchain network.

##### Registration of Smart Home Smart Contract

Step 1: The registration of the smart home is performed indirectly through the IoT controller. The$\;i^{\mathrm{th}}\;$smart home calls upon the Application Programming Interface (API) of the registered smart home (register_SH) smart contract via the$\;p^{\mathrm{th}}\;$IoT controller. The$\;i^{\mathrm{th}}\;$smart home provides the necessary information, including the $p^{\mathrm{th}}\;$IoT controller public key$\;PK_{p}^{IoT^{C}}$, smart home location$\;X_{i}^{SH},\;Y_{i}^{SH}$*,* smart home sub-area$\;SA_{i}^{SH}$, category, smart homeowner phone number$\;PH_{i}^{SH}$, temperature device identity$\;TDI_{i}^{SH}$, smoke device identity$\;SID_{i}^{SH}$, humidity device identity$\;HID_{i}^{SH}$fire alarm identity$\;FAI_{i}^{SH}$, and IoT gateway identity$\;IGI_{i}^{SH}\;$can be expressed as given by Equation (22).

(22) $$register_{SH}=<PK_{p}^{IoT^{C}}\left\|X_{i}^{SH}\right\|Y_{i}^{SH}\|SA_{i}^{SH}\|category\|\;PH_{i}^{SH}\|\;TDI_{i}^{SH}\|SID_{i}^{SH}\|HID_{i}^{SH}\|\;FAI_{i}^{SH}\|IGI_{i}^{SH}\|timestamp>$$

Step 2: The $p^{\mathrm{th}}$ IoT controller receives registration request information and signs the registration request using its private key$\;SK_{p}^{IoT^{C}}\;$and forwards it to the endorsing peer, which can be expressed as given by Equation (23).

(23) $$Tx=SK_{p}^{IoT^{C}}<register\_SH\|timestamp>$$

The endorsing peer verifies the $p^{\mathrm{th}}$ IoT controller$\;PK_{p}^{IoT^{C}}$, signs the registration request using its digital certificate$\;Cert\_Org_{peer}^{F}\;$and sends back the signed transaction to the client through Tx, which can be expressed as given by Equation (24).

(24) $$Tx=Cert\_Org_{peer}^{F}\;<register\_SH\|PK_{p}^{IoT^{C}}\|timestamp>$$

The client forwards this signed transaction to the orderer. The orderer validates the transaction information and generates a new block. This block is broadcast to the committing peer and can be expressed as given by Equation (25).

(25) $$Block=Orderer<Tx^{ID}\left\|register\_SH\right\|Cert\_Org_{peer}^{F}\|timestamp>$$

Step 3: The committing peer informs the client and returns a pair of public/private keys for the $i^{\mathrm{th}}\;$smart home IoT gateway$\;PK_{i}^{SH^{IoT\_G}},SK_{i}^{SH^{IoT\_G}}$. The IoT controller informs the $i^{\mathrm{th}}\;$smart home of successful registration and provides the same pair of keys. The IoT controller stores the public key of the $i^{\mathrm{th}}\;$smart home IoT gateway and the IoT device’s identity are in its access control list.

##### Registration of Public Emergency Service Department Smart Contract

Step1: The registration of a PES department is indirectly performed using a service controller. The$\;j^{\mathrm{th}}$ PES department calls upon the API of the registered public emergency service department (register_PESD) smart contract. The $j^{\mathrm{th}}\;$PES department sends the desired information, including the service controller public key$\;PK^{S^{C}}$*,* PES department location$\;X_{j}^{PESD},\;Y_{j}^{PESD}$, PES department sub-area$\;SA_{j}^{PESD}$, PES department valid identity $ID_{j}^{PESD}$and PES department service queue length$\;SQL_{j}^{PESD}$, which can be expressed as given by Equation (26).

(26) $$register\_PESD=<\;PK^{S^{C}}\|X_{j}^{PESD}\|Y_{j}^{PESD}\|SA_{j}^{PESD}\|\;ID_{j}^{PESD}\|SQL_{j}^{PESD}\|timestamp>$$

Step2: The service controller receives the $j^{\mathrm{th}}\;$PES department registration request information and signs it using its private key$\;SK^{S^{C}}$. The service controller forwards the signed registration request to the endorsing peer, which can be expressed as given by Equation (27).

(27) $$Tx=SK^{S^{C}}<register\_PESD\|timestamp>$$

The endorsing peer checks the received information and signs the transaction using its digital signature $Cert\_Org_{peer}^{F}\;$and returns it to the client through Tx, which can be expressed as given by Equation (28).

(28) $$Tx=Cert\_Org_{peer}^{F}\;<register\_PESD\|\;PK^{S^{C}}\|timestamp>$$

The client forwarded this signed transaction to the orderer. The orderer collects the number of signed transactions, generates a new block, and passes it to the committing peer to update their distributed ledger information, which can be expressed as given by Equation (29).

(29) $$Block=Orderer<Tx^{ID}\|register\_PESD\|Cert\_Org_{peer}^{F}\|timestamp>$$

Step3: The committing peer notifies the client about the successful registration of $j^{\mathrm{th}}\;$PES department and returns a pair of public/private keys$\;PK_{j}^{PESD},\;SK_{j}^{PESD}$. The service controller informs the $j^{\mathrm{th}}\;$PES department and forwards the same pair of keys, its public key and latest service queue length information to its local server. 

##### Call Public Emergency Service Department Service Provider Smart Contract

Step1: The $i^{\mathrm{th}}\;$smart home IoT gateway uses its private key$\;SK_{i}^{SH^{IoT\_G}}\;$and sends the IoT device data to the$\;p^{\mathrm{th}}\;$IoT controller. The $p^{\mathrm{th}}\;$IoT controller continuously monitors these smart IoT device data. When IoT devices reach the threshold, the $p^{\mathrm{th}}\;$IoT controller invokes the call public emergency service department service provider *(*call_PES_servicProvider) smart contract on behalf of $i^{\mathrm{th}}\;$smart home. $p^{\mathrm{th}}\;$IoT controller inserts the received information, such as the public key of $i^{\mathrm{th}}\;$smart home IoT gateway$\;PK_{i}^{SH^{IoT\_G}},\;\;i^{\mathrm{th}}\;$smart home location$\;X_{i}^{SH},\;Y_{i}^{SH}$*,* smart home sub-area$\;SA_{i}^{SH}$, and threshold values $Th_{i}^{\alpha },Th_{i}^{\beta },\;Th_{i}^{\gamma }\;$and the $p^{\mathrm{th}}\;$IoT controller sends the signed transaction using its private key$\;SK_{p}^{IoT^{C}}$, which can be expressed as given by Equation (30).

(30) $$call\_PESD\_serviceProvider=SK_{p}^{IoT^{C}}\;<\;PK_{i}^{SH^{IoT_{G}}}\|X_{i}^{SH}\|\;Y_{i}^{SH}\|SA_{i}^{SH}\|\;Th_{i}^{\alpha }\|Th_{i}^{\beta }\|Th_{i}^{\gamma }\|timestamp>$$

The PES request of $i^{\mathrm{th}}\;$smart home is broadcast on the private blockchain network through Fabric organisation, which can be expressed as given by Equation (31).

(31) $$Tx=Cert\_Org_{peer}^{F}\;<call\_PESD\_ServiceProvider\|timestamp>$$

Step2: The service controller retrieves the required information from transaction Tx to avail the PES provider of$\;j^{\mathrm{th}}\;$PES department with the minimum service queue length for$\;i^{\mathrm{th}}\;$smart home (see Section 4.2).

Step3: After selecting $j^{\mathrm{th}}\;$PES department, the service controller, proposes a transaction that includes an expected reachable time$\;ERT_{j,i}\;$of PES provided at $i^{\mathrm{th}}\;$smart home and a public key $PK_{j}^{PESD}\;$of selected $j^{\mathrm{th}}\;$PES department, which can be expressed as given by Equation (32).

(32) $$Tx=Cert\_Org_{peer}^{F}=<ERT_{j,i}\;\|PK_{j}^{PESD}\|timestamp>$$

The orderer receives transaction information, generates a new block and broadcasts in the private blockchain network, which can be expressed as given by Equation (33).

(33) $$Block=Orderer<Tx^{ID}\|call\_PESD\_serviceProvider\|Cert\_Org_{peer}^{F}\|timestamp>$$

The other Fabric organisation receives this information and uses it later to generate reputation value for $j^{\mathrm{th}}\;$PES department after fulfilment of the PES request.

##### Reputation Generation for Public Emergency Service Department Smart Contract

Step 1: The$\;p^{\mathrm{th}}\;$IoT controller uses its private key$\;SK_{p}^{IoT^{C}}\;$to generate either a positive or negative reputation value for the PES department by calling a call reputation generation for a public emergency service department (reputationGeneration_PESD) smart contract on behalf of $i^{\mathrm{th}}\;$smart home. The $p^{\mathrm{th}}\;$IoT controller inserts the necessary information, such as the public key of $j^{\mathrm{th}}\;$PES department$\;PK_{j}^{PESD}$, the public key of$\;i^{\mathrm{th}}\;$smart home IoT gateway$\;PK_{i}^{SH^{IoT\_G}}$,$\;PRV_{j}^{PESD}$ or $NRV_{j}^{PESD}$ for $j^{\mathrm{th}}\;$PES department, expected arrival time$\;EAT_{j}^{PESD}$and actual reaching time$\;ART_{j}^{PESD}$ of $j^{\mathrm{th}}\;$PES department (see Section 4.3), which can be expressed as given by Equation (34).

(34) $$reputationGeneration\_PESD=SK_{p}^{IoT^{C}}<\;ART_{j}^{PESD}\|\;EAT_{j}^{PESD}\|\;PK_{j}^{PESD}\|PK_{i}^{SH^{IoT\_G}}\|\;PRV_{j}^{PESD}\|NRV_{j}^{PESD}\|timestamp>$$

Step 2: This information is forwarded on the private blockchain through the Fabric organisation of the $p^{\mathrm{th}}\;$IoT controller to take further action, which can be expressed as given by Equation (35).

(35) $$Tx=Cert\_Org_{peer}^{F}\;<reputationGeneration\_PESD\|timestamp>$$

Step 3: The orderer receives the reputation information of the $j^{\mathrm{th}}\;$PES department and generates a new block to broadcast information to other Fabric organisations, which can be expressed as given by Equation (36).

(36) $$Block=Orderer<Tx^{ID}\|reputationGeneration\_PESD\|Cert\_Org_{peer}^{F}\|timestamp>$$

##### Final Reputation Update for Public Emergency Service Department Smart Contract

Step 1: At the end of the day, the service controller evaluates the final reputation value for the $j^{\mathrm{th}}\;$PES department based on the positive and negative reputation value generated by the $p^{\mathrm{th}}\;$IoT controller on behalf of the $i^{\mathrm{th}}\;$smart home. The service controller calls the final reputation update for the public emergency service department (finalReputationUpdation_PESD) smart contract. The service controller enters the necessary information such as the public key $PB_{j}^{PESD}$and final reputation $FRV_{j}^{PESD}$for the $j^{\mathrm{th}}\;$PES department generates a signed transaction using its private key $SK^{S^{C}}$, which can be expressed as given by Equation (37) (see Section 4.3).

(37) $$finalReputationUpdataion\_PESD=SK^{S^{C}}<PB_{j}^{PESD}\left\|FRV_{j}^{PESD}\right\|timestamp>$$

Step 2: The Fabric organisation of the service receives this information and forwards the signed transaction in the private blockchain network, which can be expressed as given by Equation (38).

(38) $$Tx=Cert\_Org_{peer}^{F}\;<finalRputationUpdataion\_PESD\|timestamp>$$

Step 3: The orderer processes this information to create a new block and broadcast it to other Fabric organisations, which can be expressed as given by Equation (39).

(39) $$Block=Orderer<Tx^{ID}\|finalReputationUpdataion\_PESD\|Cert\_Org_{peer}^{F}\|timestamp>$$

## 5. Simulation Results and Discussion

### 5.1. Simulation Settings 

To set up the private blockchain network for the BEST framework, we used Hyperledger Fabric 2.x. The private blockchain network is deployed on a cloud platform with 16 CPU cores, 12 GB RAM, and 125 GB storage. Further, we used Python to call upon blockchain API for IoT gateways and PES departments. We utilised a random number generator to generate an eight-digit identity for the IoT devices and IoT gateway connected with a smart home to distinguish them from other smart home IoT devices and gateways. The IoT controller stores this information in its access control list to identify the number of IoT devices linked with smart homes with their identity. With the help of an access control list, the IoT controller easily detects the placement of any malicious IoT device in a smart home. The BEST framework consists of nine Fabric organisations running on docker; among them, one is an administrator, seven are IoT controllers, and the last one is a service controller. The administrator’s task is to first deploy a private blockchain network on the cloud platform. The configtxgen tool is utilised to generate the genesis block, which contains the private blockchain network configuration and a channel. Additionally, the cryptogenic tool is used to generate the digital certificate (i.e., X.509 certificate) and public/private key for the endorsing peer, committing peer, and client residing in the Fabric organisation and for the orderer. Once the network is set up, and all credentials are generated using cryptogenic and configtxgen tools, the administrator installs all the smart contracts in the private blockchain network. 

Few assumptions are considered while designing the smart contracts, queue model, and reputation model in the BEST framework, as shown in Table 2. We considered seven IoT controllers in the BEST framework, and accordingly, we have seven sub-areas in a smart city, and the number of smart homes in each sub-area is assumed as 50, whereas each sub-area contains one PES department to handle the PES request of a smart home. Each PES department contains ten PES providers, due to which the maximum service queue length of each PES department is ten. The threshold values correspond to temperature$\;Th^{\alpha }$, smoke$\;Th^{\beta }$, and humidity$\;Th^{\gamma }\;$device are assumed as 60 °C, 120 ppm, and 65%, respectively, which indicates the presence of fire in a smart home. The values of β and γ to adjust the reputation value are considered as 0.5 and 0.014, respectively. The distance$\;D_{i,j}\;$between the$\;i^{\mathrm{th}}\;$smart home and$\;j^{\mathrm{th}}\;$PES department within a smart city lies between 5 and 50 Km, and the average speed of the $j^{\mathrm{th}}\;$PES department is considered between 50 and 60 km/h. Finally, the time duration$\;T_{i,j}\;$To reach at the$\;i^{\mathrm{th}}\;$smart home due to high traffic is adjusted between 15 and 30 min for the$\;j^{\mathrm{th}}\;$PES department.

The centralised PES system only contains the information of its sub-area PES department and its PES providers. These centralised PES systems only fulfil the PES requests of its sub-area smart homes. In an emergency, these sub-areas are unable to handle the other sub-areas PES requests because they do not have a global view of all the sub-areas smart home locations. Furthermore, a PES department within a sub-area is unable to accept a PES request of its sub-area smart home if all its PES providers are engaged, which could lead to serious fire damage in a smart city. We considered the centralised PES system for comparison with the proposed BEST framework for the state-of-the-art work.

### 5.2. Result Analysis 

**Figure 6:**
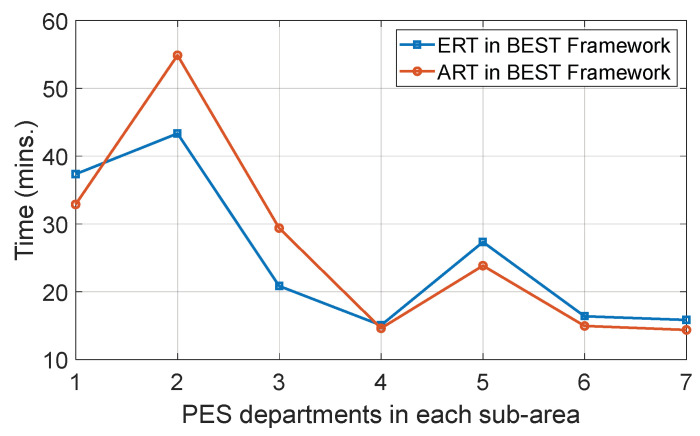
Evaluation of ERT and ART for PES departments.

A relationship between the expected reaching time$\;ERT_{j,i}\;$and the actual reaching time$\;ART_{j,i}\;$for the$\;j^{\mathrm{th}}\;$PES department to reach the$\;i^{\mathrm{th}}\;$smart home for the BEST framework is shown in Figure 6. It is observed that the PES departments of sub-area 2 and 3 are unable to reach the fire within the expected reaching time, due to which they receive a negative reputation value from the corresponding$\;i^{\mathrm{th}}\;$smart home, whereas the rest receive a positive reputation value. To evaluate the output, we assumed that in each sub-area, the$\;i^{\mathrm{th}}\;$smart home generates a PES request. Initially, the waiting time and reaching time are evaluated for the$\;j^{\mathrm{th}}\;$PES department using Equations (4) and (6), respectively. The time consumption is considered based on Table 2 data. Further, by utilising all three pieces of information, the expected reaching time is calculated using Equation (8) and the actual reaching time by varying time consumption information. 

**Figure 7:**
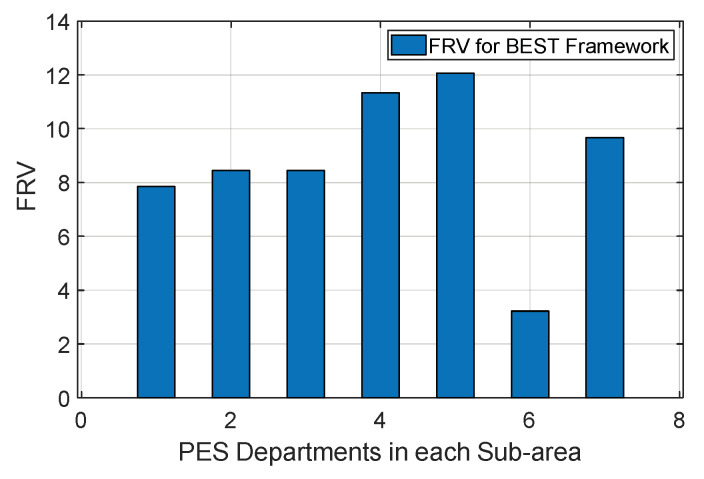
Evaluation of FRV for PES departments.

The final reputation value$\;FRV_{j,i}\;$for the$\;j^{\mathrm{th}}\;$PES department is obtained from multiple$\;i^{\mathrm{th}}\;$smart homes in the BEST framework, as shown in Figure 7. It is observed that the PES department in sub-area 1 has the maximum final reputation value because it served the maximum in-time PES requests generated from smart homes. However, the final reputation value of the PES department in sub-area 7 has the minimum value. To evaluate the output of various positive reputations$\;PRV_{j,i}\;$and negative reputations $NRV_{j,i}$, values are collected corresponding to the$\;j^{\mathrm{th}}\;$PES department using Equations (9) and (10), respectively. Further, both reputation values are considered collaboratively to identify a single final reputation value for the$\;j^{\mathrm{th}}\;$PES department using Equation (11) at the end of the day. The reputation model helps the PES departments improve their future performance by analysing their in-time and delayed PES requests fulfilled in a day. 

**Figure 8:**
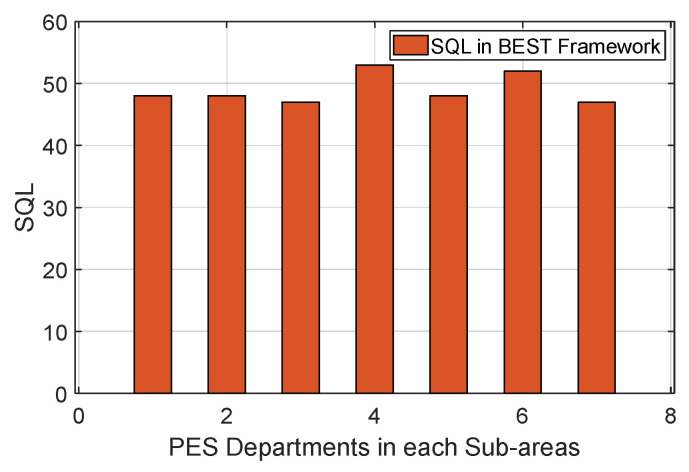
Evaluation of SQL for PES departments.

A relationship between the service queue length$\;SQL_{j}^{PESD}\;$and $j^{\mathrm{th}}\;$PES department for the BEST framework is shown in Figure 8. It is observed that the service queue length of various PES departments is nearby because the service controller is distributing the PES request load on each PES department equally. To evaluate the output, we considered a random distribution 15–20/h to generate the PES request corresponding to a smart home in a sub-area. Further, the service rate of a PES department depends on the number of PES providers it has and the frequency at which its PES providers are fulfilling the PES request. Using these two pieces of information, we evaluated the service queue length for each$\;j^{\mathrm{th}}\;$PES department using Equations (2) and (3). For instance, a new PES request is generated from the$\;i^{\mathrm{th}}\;$smart home, and the service controller first identifies the service queue length of each PES department and selects the one with the minimum service queue length based on its sub-area. In Figure 8, the PES department in sub-area 3 has a high chance of selection. 

**Figure 9:**
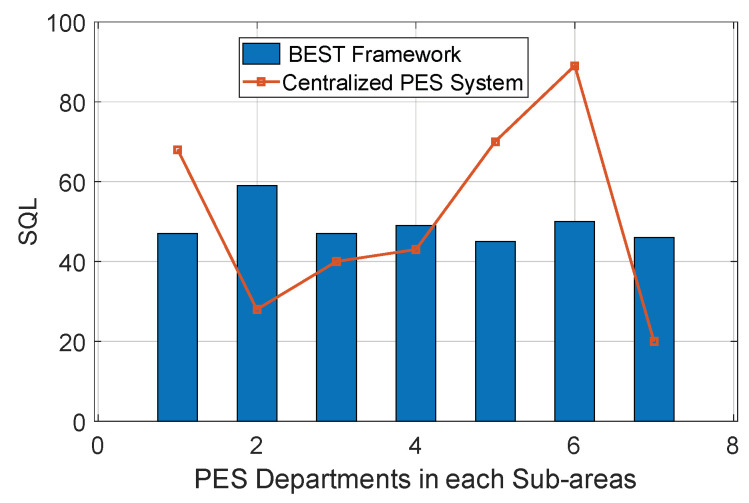
Comparison between the BEST framework and centralised PES system for SQL.

A comparison between the centralised PES system and the BEST framework concerning the service queue length is shown in Figure 9. It is observed that in the BEST framework, the PES requests are equally distributed among all PES departments based on their service queue length by the service controller. Hence, the waiting time required to confirm the PES request generated from the$\;i^{\mathrm{th}}\;$smart home is the minimum. In comparison, the centralised PES system takes maximum waiting time to confirm the PES request of the $i^{\mathrm{th}}$ smart home because there is no controller available in the centralised PES system that keeps a global view of the service queue length of each PES department. Due to this, the service queue length of the PES department in sub-areas 5 and 6 has the maximum burden. To generate the output for the BEST framework, we evaluate the service queue length$\;SQL_{j}^{PESD}\;$using Equations (2) and (3) for all PES departments. For instance, the$\;i^{\mathrm{th}}\;$smart home generates a PES request in sub-area 1, and the BEST framework selects the PES department of sub-area 5 because it has a minimum service queue length.

Similarly, in the same sub-area, a PES request is generated by the$\;i^{\mathrm{th}}\;$smart home, the centralised PES system has no option and forwards the request to the same sub-area PES department. Hence, it burdens the PES department of the centralised PES system.

**Figure 10:**
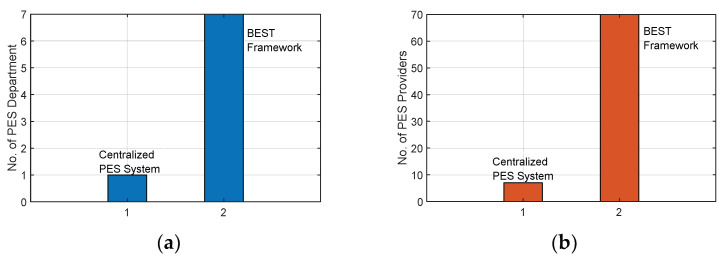
(**a**) A number of PES departments (**b**) Number of PES providers for the BEST framework and centralised PES system.

A comparison between the centralised PES system and the BEST framework with respect to the number of PES departments and the number of PES providers is shown in Figure 10a,b, respectively. Figure 10a shows that in the BEST framework, the total number of PES departments to receive the PES request is greater than the centralised PES system. In the BEST framework, PES departments of various sub-areas are connected with a service provider through the private blockchain network so that in an emergency, these PES departments fulfil the PES requests of the same sub-area and other sub-areas. However, a PES department only handles its sub-area PES request in the centralised PES system. Similarly, in Figure 10b, it is identified that the number of PES providers in the BEST framework is far more than the centralised PES department. The maximum number of PES requests handled by the BEST framework at a time equals 70, which directly depends on the number of PES providers available in the network, whereas the maximum number of PES requests is processed by the centralised PES system is only 10. 

**Figure 11:**
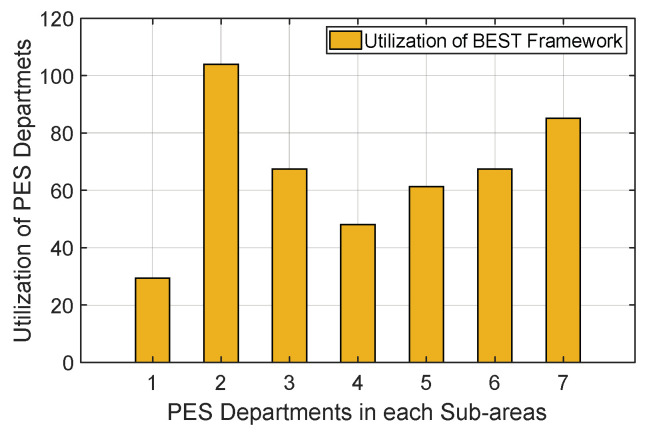
Evaluation of the utilisation of PES departments.

A relationship between the utilisation of$\;U_{j}^{PESD}\;$ and the $j^{\mathrm{th}}\;$ PES department for the BEST framework is shown in Figure 11. It is observed that the PES department in sub-area 7 has the maximum utilisation because most of its PES providers are engaged in fulfilling PES requests for $i^{\mathrm{th}}\;$smart homes located in the same or different sub-areas.

In comparison, the utilisation of the PES department available in sub-areas 3 and 6 are low and nearly the same. This happens when this PES provider of the selected$\;j^{\mathrm{th}}\;$ PES department is unable to fulfil its request within the desired time. To evaluate the output, we considered a random distribution [6–10]/h to generate the PES requests and a random service rate between 15 and 30 min for each PES department. This information is used to generate the utilization $U_{j}^{PESD}\;$for the $j^{\mathrm{th}}\;$ PES department using Equation (1). 

**Figure 12:**
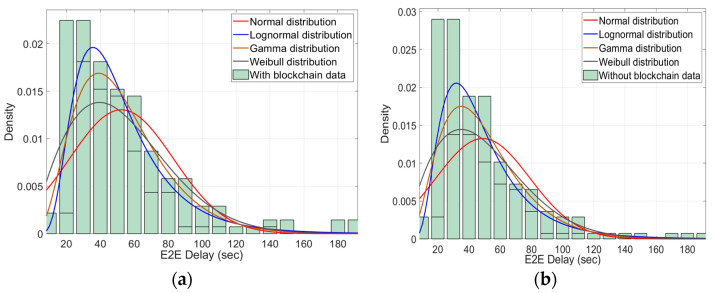
(**a**): E2E delay for the BEST framework; (**b**) E2E delay for the centralised PES system.

A comparison between the End-to-End (E2E) delay for the proposed BEST framework and the centralised PES system is shown in Figure 12a,b, respectively. It is observed that the normal distribution of the BEST framework is slightly low as compared to the centralised PES system. This is because all the entities such as IoT controller, service controller, and PES departments are connected through the same private blockchain network, due to which the request and response time is the minimum. In comparison, all entities in the centralised PES system are not on the same network due to a slight delay in request and response time. To obtain the output, we considered a variable number of smart home PES requests, recorded their E2E delay and applied the distribution to view the behaviour of the BEST framework and centralised PES system under the same number of PES requests. The E2E delay is a sum of request time and response time. In the BEST framework, the request time and response time are evaluated on the private blockchain network. The request time is a timestamp between the$\;i^{\mathrm{th}}\;$smart home IoT gateway sends the threshold values, and the$\;p^{\mathrm{th}}\;$IoT controller, which generates a PES request corresponding to the $i^{\mathrm{th}}$ smart home. The response time is a timestamp stamp between the service controller that receives the PES request and the selection of the$\;j^{\mathrm{th}}\;$PES department to fulfil the PES request. In the centralised PES system, there is no request and response time, and a direct communication occurs between the smart home IoT gateway and the IoT controller, and between the IoT controller and the PES department.

## 6. Conclusions and Future Work

The blockchain holds the promises of transparency, trust, and privacy for an IoT-based smart city. Therefore, applying blockchain directly to IoT networks is not a good option because of numerous challenges, including resource consumption, processing time, storage, and scalability. In this paper, we proposed a three-layered architecture of BEST that help in providing reliable PES. In the proposed system architecture, the IoT controller uses a queue model to provide fast access to PES providers. The benefit of an IoT controller is off-chain storage, proper management of IoT devices through an access control list, and scalability. In contrast, the queue model helps in selecting an appropriate PES department. The private blockchain network of the BEST framework is designed using a Hyperledger Fabric platform, which maintains records of IoT controllers, service controllers, smart homes and PES departments in the distributed ledger. The transfer of PES requests and the arrival of PES providers are ensured by using smart contract implementation. We also considered the reputation model for the PES department. The smart home rates the PES department according to their service fulfilment and generates either a positive or negative reputation value accordingly. The results indicate that our system model is sufficient to handle PES requests in real-time and ensure minimum waiting to fulfill a PES request. As a limitation of this study, it is noted that a simulation-based experimental study needs further validation in hardware-based prototype testing. We will develop Raspberry-pi-enabled blockchain nodes for prototype validation of the BEST framework. In the future, we will extend our work by utilising neuro-fuzzy logic to identify the presence of fire in a smart home. A hybrid blockchain platform will also be a quest for the implementation of several smart contracts as well as using the incentive mechanism for PES departments to incentivise them based on their reputation value after fulfilling a PES request.

## Figures and Tables

**Table 1:** Abbreviation.

Symbols	Abbreviations
$U_{j}^{PESD}$	Utilisation of $j^{\mathrm{th}}\;$PES department
$\lambda_{j}^{PESD}$	Arrival time of PES requests at $j^{\mathrm{th}}$ PES department
$\mu_{i}^{PESD}$	Service rate of $j^{\mathrm{th}}$ PES department
$SQL_{j}^{PESD}$	Service queue length of $j^{\mathrm{th}}$ PES department
$PI_{j}^{PESD}$	Probability of idleness of $j^{\mathrm{th}}$ PES department
$N^{PESD}$	Number of PES departments
$N^{SH}$	Number of smart homes
$N^{IC}$	Number of IoT controllers
$SA_{i}^{SH}$	Sub-area of $i^{\mathrm{th}}$ smart home
$SA_{j}^{PESD}$	Sub-area of $j^{\mathrm{th}}$ PES department
$D_{j,i}$	Distance between $i^{\mathrm{th}}$ smart home and $j^{\mathrm{th}}$ PES department
$RT_{j,i}$	Reaching time for $j^{\mathrm{th}}$ PES department to $i^{\mathrm{th}}$ smart home
$RV_{j,i}$	Reputation value for $j^{\mathrm{th}}$ PES department generated from $i^{\mathrm{th}}$ smart home
$ERT_{j,i}$	Expected reaching time for $j^{\mathrm{th}}$ PES department to $i^{\mathrm{th}}$ smart home
$T_{j,i}$	Time duration consumed by $j^{\mathrm{th}}$ department to reach $i^{\mathrm{th}}$ smart home
$PRV_{j,i}$	Positive reputation value for $j^{\mathrm{th}}$ PES department obtained from $i^{th}$ smart home
$NRV_{j,i}$	Negative reputation value for $j^{\mathrm{th}}$ PES department obtained from $i^{\mathrm{th}}$ smart home
$FRV_{i}$	Negative reputation value for $j^{\mathrm{th}}$ PES department

**Table 2:** Parameter settings.

Parameters	Value
Sub-areas in a smart city	7
Smart homes in each sub-area	50
IoT Controllers	7
Service Controller	1
PES departments	7
PES provider in each PES department	10
Maximum service queue length of each PES department	10
b	0.5
γ	0.014
$\;Th^{\alpha },$ $Th^{\beta }$ $,\;Th^{\gamma }$	60 °C, 120 ppm, 65%
Distance$\;D_{i,j}$ between the$\;i^{\mathrm{th}}\;$smart home and$\;j^{\mathrm{th}}\;$PES department	5 to 50 km
Time duration$\;T_{i,j}\;$for$\;j^{\mathrm{th}}\;$PES department in high traffic to reach $\;i^{\mathrm{th}}\;$smart home	15 to 30 min
Time interval T	24 h
Average speed$\;AS_{j}^{PESD}\;$of$\;j^{\mathrm{th}}\;$PES department	50 to 60 km/h

## Data Availability

Research data will be available on individual requests to the corresponding author considering collaboration possibilities with the researcher or research team and with restrictions that the data will be used only for further research in the related literature progress.
